# Adipocire—Its Formation

**Published:** 1855-09

**Authors:** 


### ADIPOCIRE—ITS FORMATION.

Dr. CHARLES M. WITHERILL read last winter an able memoir on this subject before the American Philosophical Society, which has been published in the Society's Transactions. The author has performed a number of experiments tending to determine the manner in which adipocire is formed and the materials which go to constitute it. The general belief among chemists since this substance was first carefully examined by Fourcroy, has been that it is formed out of all animal substances except heair, nails and bones. Lord Bacon affirms in one of his works, that nearly all flesh may be turned into a fatty substance by cutting it into pieces and putting it into a glass covered with parchment, then letting the glass stand six or seven hours in boiling water. In 1786–7 the Cemetiere des Innocens, at Paris, having been opened for the removal of the bodies to another grave yard, Fourcroy had ample means of observing the circumstances under which adipocire is formed. He found an abundance of the substance, especially in the ditches, where the slightly made coffins of the poorer classes had been piled one upon another; the trench being open for some time till it was filled with bodies when it was slightly covered with earth. On opening the trenches after some fifteen years the bodies were converted into adipocire; they were flattened by mutual pressure and had impressions on their surface of the grave clothes. George Smith Gibbes, a few years afterwards, observed that in Oxford, in the pits where were thrown the remains of dissections, and at the bottom of which flowed a gentle current of water, large quantities of adipocire were formed; and placing a piece of beef in the river in a box pierced with holes, this substance also resulted. He states that nitric acid will effect the same change in a few days. Bostock seemed to prove by some experiments which he instituted, that muscular fibre could be converted into fat by the action of nitric acid. Chevreul, on repeating his experiments, could not obtain fat from pure fibrine; but Von Bibra, as the result of many experiments, concluded that muscle, under certain circumstances, is changed to fat, and Blondeau arrived at the same conclusion from an examination of the cheese manufacture at Roquefort. The cheese when first made contained 1·200 of its weight of fat, but after two months in dark, cool, damp cellars, the caseine was almost wholly converted into fat. A piece of beef, after two months in the cellar, surrounded with paste and slightly salted, was found changed, for the greater part, into a fatty body, presenting the greatest analogy to hog's lard.

The question is an interesting one in a physiological point of view. Some experiments recently performed by Liebig, Bopp, Keller and others, rendered possible the formation of fat from albumen, fibrin and caseine. It is certain that herbivorous animals possess more fat than is taken in their food, but it has generally been conceded that the excess comes from the sugar and other non-nitrogenized compounds. In like manner where organs in the animal body have undergone the fatty degeneration, the oil is supposed to be derived from other parts of the system, rather than formed in the part by a metamorphosis of the nitrogenous compounds.

Dr. Witherill engaged in the study of adipocire with reference to this question. In his investigations he found that this substance seemed to be formed under opposite circumstances. In some graves it was discovered, and in others contiguous to them decomposition had advanced to its full extent, leaving nothing but the skeleton. The preservation of some bodies seems, indeed, inexplicable in the present state of our knowledge, as, for example, the case of General Washington, who, having reposed in his tomb for more than forty years, was so perfectly preserved as to have been recognized from the resemblance of his portraits. When Dr. Witherill engaged in the examination of adipocire he was inclined to the belief that it was a result of the blood-forming substances, but his experiments have brought him to a different conclusion, namely, that the higher members of the series of fatty acids do not result from the putrefaction of proteine compounds; and as corroborative of this view he mentions, that in all cases where adipocire has been found, the corpse was of a large and fat person. In grave-yards, he has remarked, if the proportion of flesh to fat be large, and especially if the nature of the ground be such as to prevent the escape of the decomposed matter, as by draining, no adipocire is formed, but the fat undergoes full decomposition. The microscope sustains his experiments, inasmuch as it fails to trace any arrangement of the fatty particles into fibres or rows, such as would here and there be seen if the fat came from muscles. The original fat of the body, Dr. Witherill concludes, according to circumstances after burial, either partakes of a decomposition by which its elements escape, or else, losing its glycerine and most of its oleic acid, becomes gradually converted into adipocire. We do not suppose that his experiments settle the question, but to our mind they are nearly conclusive.

